# Comparing the Effectiveness of Malaria Vector-Control Interventions Through a Mathematical Model

**DOI:** 10.4269/ajtmh.2010.09-0179

**Published:** 2010-08-05

**Authors:** Nakul Chitnis, Allan Schapira, Thomas Smith, Richard Steketee

**Affiliations:** Department of Public Health and Epidemiology, Swiss Tropical and Public Health Institute, Basel, Switzerland; MACEPA, PATH, Ferney-Voltaire, France

## Abstract

Although some malaria-control programs are beginning to combine insecticide-treated nets (ITNs) and indoor residual spraying (IRS), little is known about the effectiveness of such combinations. We use a mathematical model to compare the effectiveness of ITNs and IRS with dichlorodiphenyltrichloroethane (DDT) or bendiocarb, applied singly and in combination, in an epidemiological setting based in Namawala, Tanzania, with *Anopheles* *gambiae* as the primary vector. Our model indicates that although both IRS (with DDT) and ITNs provide personal protection, humans with only ITNs are better protected than those with only IRS, and suggests that high coverage of IRS with bendiocarb may interrupt transmission, as can simultaneous high coverage of ITNs and IRS with DDT. When adding a second vector-control intervention, it is more effective to cover the unprotected population first. Although our model includes some assumptions and approximations that remain to be addressed, these findings should be useful for prioritizing and designing future field research.

## Introduction

Mathematical modeling can play an important role in quantifying the effects of malaria-control interventions and determining efficient combinations of these interventions. We have published a deterministic dynamical systems model (Appendix Box 1 has a glossary of modeling terms) to describe the dynamics of malaria in a mosquito population interacting with a heterogeneous population of humans.^1^ The purpose of the present paper is 2-fold: to explain this model to a non-mathematical audience and to use it for exploring the impact of combinations of insecticide-treated nets (ITNs) and indoor residual spraying (IRS). We assume that ITNs are treated with a pyrethroid, and we model IRS with either dichlorodiphenyltrichloroethane (DDT) or bendiocarb. DDT is highly repellent, and bendiocarb has almost no repellency effects; therefore, they provide two extreme examples of commonly used insecticides for IRS.

We emphasize that this work is theoretical and early, containing a number of assumptions on mosquito behavior and malaria transmission, including assuming the presence of only one vector, *Anopheles gambiae*, and ignoring seasonality and transient effects such as the decay of nets and insecticide. These assumptions are reviewed in Discussion. It will be necessary to adapt the current model to scenarios including more realistic distributions of vector species, seasonality, and transient dynamics and to calibrate assumptions and parameters against more field data before the predictions could be included as evidence for formulating malaria-control strategies. Additionally, the model described here forms part of a broader project for developing a stochastic agent-based model for malaria epidemiology and immunology, aiming to describe the impact of combinations of interventions at different coverage levels on human morbidity and mortality in realistic epidemiological and health-system scenarios.^2^ Nonetheless, we consider that, at this stage, a presentation of the deterministic mosquito transmission model should be helpful for stimulating the vital interaction between field research, modeling, and program planning.

The mathematical details and analysis of this model are in ref. 1. It is based on the model by Saul and others^3^ that describes the mosquito feeding cycle, which was further developed by refs. 4–6. The model is a system of difference equations for the number of infectious, infected, and total mosquitoes seeking for blood in a given area. We choose difference equations, because a time step of 1 day better captures the temporal dynamics of the mosquito life and gonotrophic cycle than continuous time. The population of mosquitoes is assumed to be perfectly mixing, although it would be possible to model different subpopulations or species of mosquitoes by replicating the system of difference equations with different parameter values.

This model of mosquito survival and malaria transmission^1^ does not include the effects of the malaria cycle in humans. To model the relationship of the human infectivity to mosquitoes to the entomological inoculation rate (EIR), we use equilibrium results from Killeen and others.^7^ In subsequent work, we will investigate the transient behavior of the full malaria cycle by integrating the mosquito model with human intra-host simulation models.^2^

Although our model is general enough to allow the human population to be differentiated down to an individual level,^1^ here we model the intervention strategies by dividing the human population into groups that either have the intervention or do not have it. These groups are assumed to be homogeneous, and so, all humans in each group are assumed to be identical. Also, although we do not include animal hosts in the simulations in this paper, our model can allow for various types of hosts (i.e., blood-meal sources) and thus, include the effects of zooprophylaxis.^4^,^6^ We also do not present results of comparing novel interventions such as entomopathogenic fungi or transgenically modified mosquitoes. Although these interventions may have important roles to play in integrated vector-management strategies,^8^ we now choose to focus on interventions that are currently in widespread use by national malaria-control programs. However, we hope to soon extend our model to include the effects of other such strategies.

In the next section, we describe the details of our model.^1^ We then show numerical simulations of three intervention strategies, followed by our interpretation of the results. In the Appendix, we describe the mathematics for these simulations and our data sources for the parameter values.

## Model Description

### Vector-feeding cycle.

**Figure 1.**
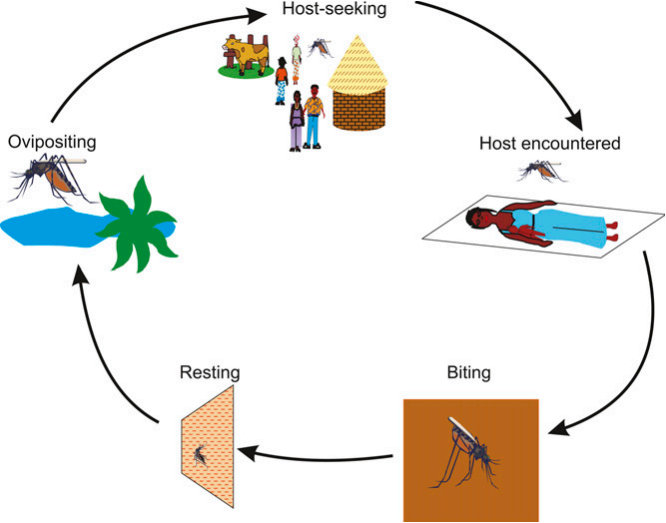
Cartoon of mosquito feeding cycle. The feeding (or gonotrophic) cycle of the female mosquito vector. After emergence, mosquitoes seek and bite hosts, rest, and lay eggs before seeking hosts again. The mosquito experiences varying levels of risk in each state. Modified from Figure in ref. 1. This figure appears in color at www.ajtmh.org.

After emergence from a breeding site, mosquitoes mate, and the females search for blood meals that are necessary for egg development. After encountering and biting a host, the female mosquito finds a resting place where it digests the blood and evaporates water. The resting time is temperature-dependent (shorter at higher temperatures) and is usually 2–3 days in tropical areas. After digesting the blood, the mosquito flies in search of a water body to lay the eggs before seeking a host again to repeat the feeding cycle. Figure 1 shows a cartoon of the feeding cycle. Usually, mosquitoes begin host-seeking at the same time every night. If they are unsuccessful in biting, they rest through the day and try again the next night. The probability that a mosquito is successful in completing a feeding cycle depends on a variety of factors, including whether the available human hosts are protected by ITNs or IRS.

**Figure 2.**
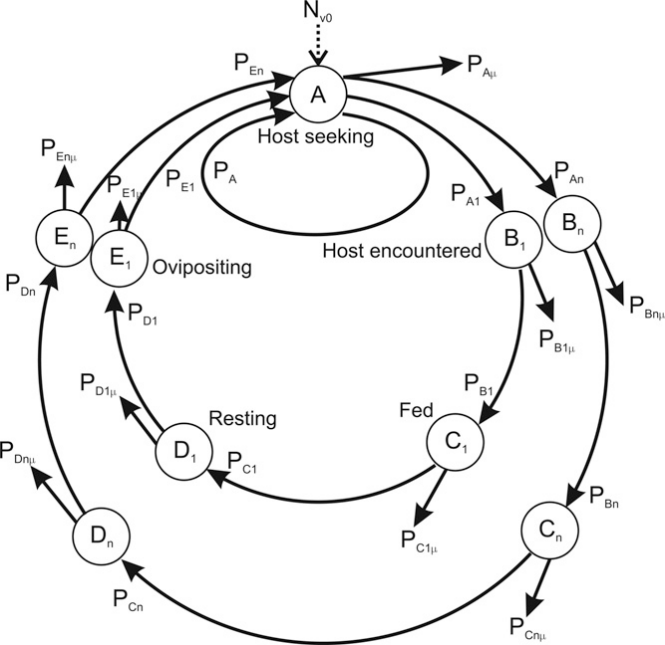
Schematic describing the processes in the feeding cycle of the female mosquito. New mosquitoes emerge from water bodies (and mate) at rate *N_v_*_0_ into the host-seeking state *A*, where they actively search for blood meals. A mosquito may encounter and feed on up to *n* different types of hosts. Each type of host, represented by subscript *i* for 1 ≤ *i* ≤ *n*, is available to mosquitoes at rate α*_i_*. If a mosquito does not encounter a host in a given night, it waits in the host-seeking phase until the next night, with probability, *P_A_*. When a mosquito encounters a host of type *i* and is committed to biting the host, it moves to state *B_i_*. If the mosquito bites, it moves to state *C_i_*, where it searches for a resting place. If it finds a resting place, it moves to state *D_i_*, where it rests for τ days. After resting, the mosquito moves to state *E_i_*, where it seeks to lay eggs. If it is successful in laying eggs, it returns to host-seeking state, *A*, where it may then encounter any type of host. At each state, the mosquito may die with some probability, labeled by subscript μ. Reproduced, with permission, from figure 2 in ref. 1.

We model each feeding cycle of the mosquito as shown in Figure 2 where an adult female mosquito can be in one of five states (E). Four of these states (B–E) depend on the type of host on which the mosquito feeds. We label these states with a subscript *i* in 1 ≤ *i* ≤ *n*, where *i* denotes the type of host and *n* is the total number of different types of hosts. We first describe these states and the processes by which the mosquitoes enter and exit the states.

(1) State *A* is host-seeking. In state *A*, the mosquito is actively searching for a blood meal. We assume that a fixed number of mosquitoes, *N_v_*_0_, emerge every day into the total mosquito population and actively seek blood meals.
  Although we use a discrete time model for malaria in mosquitoes, we embed a continuous time model for the host-seeking phase of mosquitoes. We assume that a mosquito has a constant per-capita death rate of μ*_vA_* while host-seeking. *N_i_* is the total population of hosts of type *i*. Every host of type *i*, is available to mosquitoes at a rate α*_i_*, which depends on the type of host and the mosquito species. Mosquitoes encounter hosts of type *i* at rate α*_i_N_i_*. This rate includes any reductions in host availability because of diversionary effects; for example, a host with a diversionary intervention like a net is modeled as having reduced α*_i_*. Thus, diversion is modeled in the same way as by Killeen and Smith^6^ and Saul^4^ (as described in detail in ref. 1).
  Mosquitoes only spend a certain amount of time, θ*_d_*, searching for a blood meal per night. During this time, they can either encounter a host of type *i* and move to state *B_i_* (with probability 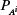), die (with probability *P_A_*_μ_), or survive but fail to find a host and remain in state *A* until the next night (with probability *P_A_*).
(2) State *B_i_* is encountering a host of type *i*. The mosquito encounters, and is committed to biting, a host of type *i*. From state *B_i_*, the mosquito can either bite the host with probability 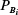 and move to state *C_i_* or die while attempting to bite and leave the population with probability 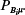.
(3) State *C_i_* is searching for a resting place. The mosquito has bitten a host of type *i* and is searching for a resting place. The mosquito can either find a resting place and move to state *D_i_* with probability 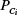 or die after biting with probability 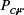.
(4) State *D_i_* is resting. The mosquito is resting after biting a host of type *i*. We assume that the mosquito rests for a fixed number of days while it digests the blood and develops eggs. It can survive this state with probability 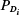 and move to state *E_i_* or die while resting and leave the population with probability 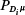.
(5) State *E_i_* is oviposition. The mosquito is seeking to lay eggs after having bitten a host of type *i*. We assume that the mosquito is able to successfully find an oviposition site, lay eggs, and return to the host-seeking state, *A*, with probability 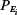 or die while trying to do so with probability 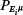.

The probability that a mosquito finds a host on a given day and then survives the feeding cycle, *P_df_*, is the product of the probabilities of surviving each stage of the feeding cycle, summed over all the different types of host that the mosquito can feed on. In our current model, this probability, *P_df_*, is the same for newly emerged mosquitoes and those that have completed one or more feeding cycles.

We let τ be the time it takes a mosquito to return to host-seeking, *A*, after it has encountered a host, *B_i_* (provided that the mosquito is still alive). This is the partial duration of the feeding cycle: it is the time that it takes a mosquito to complete a feeding cycle, excluding the time it needs to find a host from when it starts host-seeking.

### Parasite infection in the vector.

Humans infected with malaria are infective to mosquitoes if they have gametocytes in their blood. If a mosquito feeds on any human of host type *i*, there is a probability, *K_vi_*, that the mosquito will ingest both male and female gametocytes and that they will fuse in the mosquito's stomach to form a zygote, which develops into an oocyst after some temperature-dependent time, θ*_o_* (usually 3–5 days). Based on the assumption that all mosquitoes that develop oocysts will subsequently develop sporozoites to become infective, the proportion of wild-caught mosquitoes that either already have oocysts or will develop oocysts after surviving for θ*_o_* days (the delayed oocyst rate) is approximately equal to the proportion of mosquitoes that were infected when caught.^9^ After some more days, the oocysts release sporozoites that travel to the mosquito's salivary glands, and the mosquito becomes infective to humans. The temperature-dependent time that it takes an infected mosquito to become infective (be sporozoite-positive) is the extrinsic incubation period, θ*_s_* (usually 10–12 days in tropical areas). We label the probability that a host-seeking, uninfected mosquito finds a host, feeds, gets infected, and survives a feeding cycle by *P_dif_*. In our current model, this probability, *P_dif_*, is the same for newly emerged mosquitoes and those that have completed one or more feeding cycles.

### Field-measurable quantities and derived parameters.

For this model, we define several field-measurable quantities and derived parameters.^1^ These provide measures of malaria transmission that can be used to calibrate the model and determine the effectiveness of vector-control interventions. Expressions for these quantities are shown in the Appendix.

#### Parous rate.

This is the proportion of mosquitoes that have blood-fed at least one time. In this model with constant parameters (absence of seasonality), it is equal to the probability of a mosquito completing a feeding cycle, thus providing a measure of the effects of an intervention on the mosquito's mortality. We note that the parous rate is not actually a rate with units of time^−1^ but a proportion.

#### Delayed oocyst rate.

This is the proportion of infected mosquitoes. It measures the effectiveness of malaria-control interventions in reducing malaria transmission to mosquitoes. We note that the delayed oocyst rate is not actually a rate with units of time^−1^ but a proportion.

#### Sporozoite rate.

This is the proportion of infectious mosquitoes. It measures the effectiveness of control interventions in reducing malaria transmission to mosquitoes and preventing them from living long enough to become infectious. We note that the sporozoite rate is not actually a rate with units of time^−1^ but a proportion.

#### Host-biting rate.

This is the number of mosquito bites that a human host receives per day (or per other unit of time). It measures the effectiveness of control interventions in reducing mosquito bites.

#### EIR.

This is the number of infectious mosquito bites that a human host receives per day (or per other unit of time). It is the primary measure of malaria transmission.

#### Vectorial capacity.

Although not directly field-measurable, the vectorial capacity measures the ability of the mosquito population to transmit malaria, originally defined by Garrett-Jones and Grab^10^ as the “average number of inoculations with a specified parasite, originating from one case of malaria in unit time, that a vector population would distribute to man if all the vector females biting the case became infected.” The vectorial capacity measures the potential of the mosquito population to transmit malaria, not the actual transmission level.

### A short description of the mathematical transmission model.

Here, we summarize the model developed and analyzed in ref. 1 by describing the relationships instead of presenting the equations. The parameters of the model are described in Table 1, and some important derived parameters are in Table 2. We reproduce the main equations in the Appendix, but interested readers are encouraged to see ref. 1 for the mathematical details.

The system of difference equations consists of three state variables.

1. *N_v_*(*t*) is the total number of host-seeking mosquitoes on a given day, *t*.
2. *O_v_*(*t*) is the number of infected host-seeking mosquitoes (delayed oocyst positive) on a given day, *t*.
3. *S_v_*(*t*) is the number of infectious host-seeking mosquitoes (sporozoite positive) on a given day, *t*.

We note here that *S_v_*(*t*) is less than or equal to *O_v_*(*t*), which is less than *N_v_*(*t*).

The total number of host-seeking mosquitoes, *N_v_*(*t*), on a given day, *t*, is the sum of

• the new mosquitoes that emerge on that day, *N_v_*_0_;
• the host-seeking mosquitoes from the previous day that survived but were unable to find a blood meal, *P_A_N_v_*(*t* − 1);
• and the host-seeking mosquitoes from τ days earlier that successfully found a host, fed, and completed the feeding cycle, *P_df_N_v_*(*t* − τ).

The number of infected host-seeking mosquitoes, *O_v_*(*t*), on a given day, *t*, is the sum of

• the uninfected mosquitoes from τ days earlier that successfully fed, survived a feeding cycle, and got infected, *P_dif_*(*N_v_*(*t* − τ) − *O_v_*(*t* − τ));
• the infected mosquitoes from the previous day that survived but were unable to find a blood meal, *P_A_O_v_*(*t* − 1);
• and the infected mosquitoes from τ days earlier that successfully found a host, fed, and completed the feeding cycle, *P_df_O_v_*(*t* − τ).

The number of infective host-seeking mosquitoes, *S_v_*(*t*), on a given day, *t*, is the sum of

• the uninfected mosquitoes from at least θ*_s_* days ago that got infected, survived, and are host-seeking as infective mosquitoes for the first time on day *t*;
• the infective mosquitoes from the previous day that survived but were unable to find a blood meal, *P_A_Sv*(*t* − 1);
• and the infective mosquitoes from τ days earlier that successfully found a host, fed, and completed the feeding cycle *P_df_S_v_*(*t* − τ).

This system of equations represents the evolution of the mosquito population (total, infected, and infective) over time. We showed that, depending on the parameter values, there is an equilibrium set of values for the state variables, *N_v_*(*t*), *O_v_*(*t*), and *S_v_*(*t*), that the solution to this system approaches (as described in more detail in the Appendix). For parameter values representing different levels of coverage of malaria-control interventions, we can evaluate the equilibrium values of the field-measurable quantities that measure malaria transmission.

### Effects of EIR on human infectivity to mosquitoes.

The human infectivity to mosquitoes, *K_vi_*, is the result of complex processes that depend on the prevalent EIR, age, and immunity profiles of the human population and the use of antimalarial drugs. Any intervention strategies that reduces the EIR would have nonlinear effects through an increase or decrease in the human infectivity to mosquitoes.

**Figure 3.**
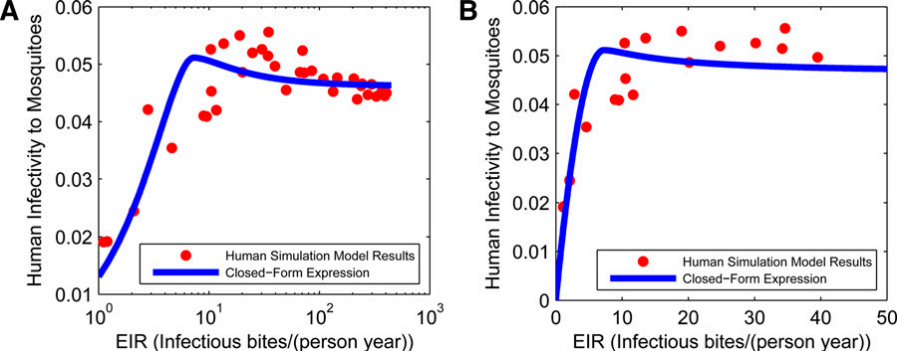
Human infectivity to mosquitoes as a function of EIR. (**A**) EIR on a logarithmic scale. (**B**) Low EIR on a linear scale. Human simulation model results from Killeen and others^7^ for human infectivity to mosquitoes, *K_vi_*, as a function of EIR and a least-squares fit of a closed-form expression approximation to these simulations. Each simulation result value of the human infectivity to mosquitoes is averaged over the human population over 1 year. The details of the closed-form expression are given in the Appendix. This figure appears in color at www.ajtmh.org.

As our current model for malaria transmission in mosquitoes^1^ does not include the malaria cycle in humans, we use the results of a stochastic simulation model^11^ to incorporate these effects at equilibrium. Killeen and others^7^ provide model simulations of the human infectivity to mosquitoes as a function of EIR, averaged over the human population over 1 year. We fit a closed-form expression for a function to these model results as described in the Appendix. The simulation results from ref. 7 and the fitted curve are shown in Figure 3.

Thus, we have, at equilibrium, the EIR as a function of the human infectivity to mosquitoes, entomological factors, and intervention coverage from the transmission model^1^ and the human infectivity to mosquitoes as a function of EIR and other factors such as acquired immunity from ref. 7. To determine the resulting EIR for a given coverage level of any intervention, we first use the transmission model to calculate the equilibrium EIR for a reasonable estimate of *K_vi_*. We then use the function (equation 5 in the Appendix) to calculate *K_vi_* for that EIR and so on until we reach convergence, allowing us to model the nonlinear effects, at equilibrium, of vector-control interventions through the malaria cycle between mosquitoes and humans.

### Numerical simulations.

We base our numerical simulations on the epidemiological setting of Namawala, Tanzania, with a pre-intervention EIR of 414 infectious bites per person per year. This EIR value is a model result derived from baseline parameter values shown in Table 3 and described in the Appendix. The effects of interventions on parameters are shown in Table 4 and described in the Appendix. We use the model to compare three malaria vector-control interventions used singly and in combination:

#### ITNs.

ITNs are the use of insecticide-treated nets, where we assume coverage and net effectiveness to be uniform over time. We base parameter values for the effects of ITNs on Killeen and Smith.^6^

#### IRS-DDT.

IRS-DDT is the use of indoor residual spraying with DDT, where we assume coverage and insecticide effectiveness to be uniform over time. We base parameter values for the effects of IRS with DDT on data from Smith and Webley^12^ from Tanzania.

#### IRS-BC.

IRC-BC is the use of IRS with bendiocarb, where we assume coverage and insecticide effectiveness to be uniform over time. We base parameter values for the effects of IRS with bendiocarb on data from Sharp and others^13^ from Bioko Island.

We model the application of each intervention, as in ref. 1. When determining the effects of an intervention used singly, we break the human population into two groups, *N* = 2, with one group representing the protected population and the other representing the unprotected population. We use the equations for the entomological quantities defined at the unique fixed point of the system of difference equations describing the dynamics of malaria in mosquitoes to determine these entomological quantities at a given coverage level. Changing the relative population sizes of each group (varying *N*_1_ and *N*_2_ for constant *N*_1_ + *N*_2_), we can determine the change in the entomological quantity as a function of coverage level. We do not allow for variation in effect over time. Thus, for example, we do not consider different durations of the effectiveness of different insecticides nor do we consider possible development of resistance.

We do not differentiate between usage and coverage. This distinction is most applicable to ITNs, because unless people who are covered by IRS replaster, wash their walls, or sleep outside, they will remain users. The parameters for ITN users reflect the reduction in mosquito biting and survival because of the properties of the nets, the biting and resting habits of the predominant Anopheline species, and the average ITN usage of the covered population.

**Figure 4.**
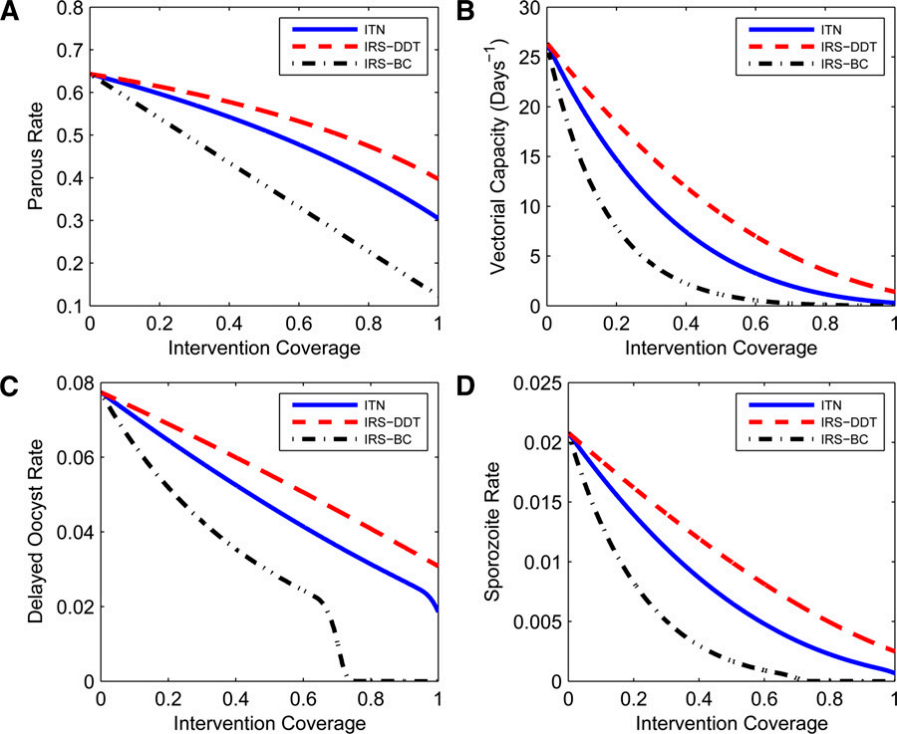
Effects of three intervention strategies, applied singly, on entomological quantities that measure mosquito survival and potential to transmit malaria in an epidemiological setting based on Namawala, Tanzania, with baseline and intervention-modified parameter values shown in Tables 3 and 4 and described in the Appendix. The plots for the delayed oocyst rate and sporozoite rate show that coverage over 80% of IRS with bendiocarb interrupts transmission. (**A**) The parous rate, as a function of intervention coverage, measures the probability of a mosquito surviving each feeding cycle. (**B**) The vectorial capacity, as a function of intervention coverage, measures the potential of the mosquito population to transmit malaria. (**C**) The delayed oocyst rate, as a function of intervention coverage, is the proportion of mosquitoes that are infected. (**D**) The sporozoite rate, as a function of intervention coverage, is the proportion of mosquitoes that are infectious to humans. This figure appears in color at www.ajtmh.org.

**Figure 5.**
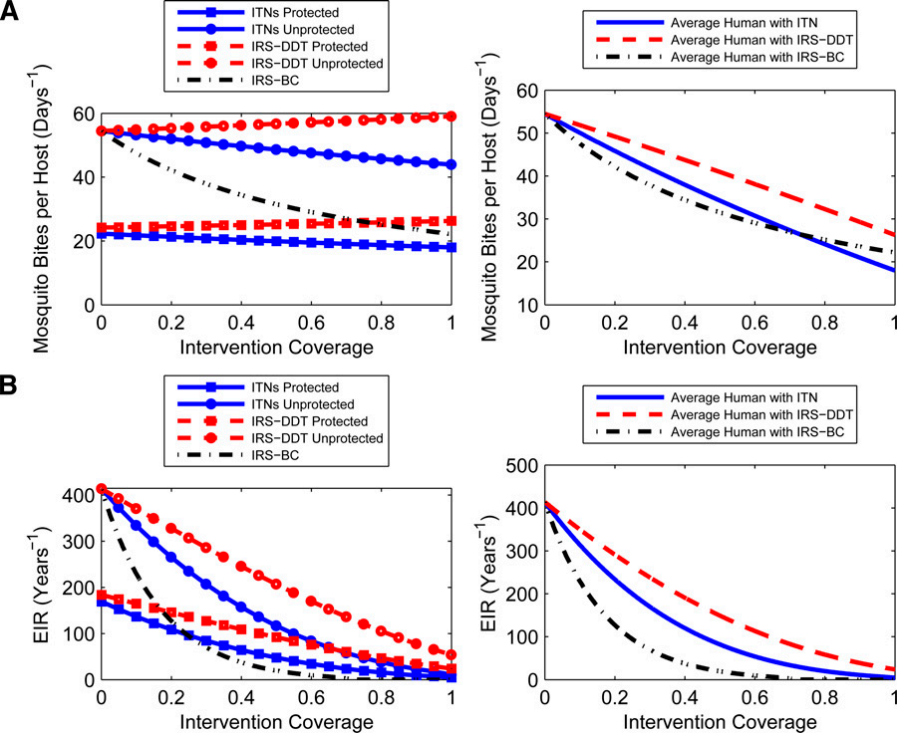
Effects of three intervention strategies, applied singly, on the host-biting rate and EIR in an epidemiological setting based on Namawala, Tanzania, with baseline and intervention-modified parameter values shown in Tables 3 and 4 and described in the Appendix. The plots on the right represent an average member of the human population. The plots on the left show the human population divided into two groups: the curves with squares represent the humans that are protected by a malaria-control intervention, and curves with circles represent the unprotected humans in a population partially protected by a malaria-control intervention. Because IRS with bendiocarb does not provide personal protection, the protected and unprotected humans have the same EIR and host-biting rate, and therefore, we only show one curve. The intervention coverage does not start at 0 but slightly above 0; where the curves appear to touch the *y* axis, one individual is protected. (**A**) The host-biting rate, as a function of intervention coverage, measures the number of mosquito bites per person per day. Note that for IRS with DDT, while the host-biting rate increases for both protected and unprotected humans as coverage increases, since the proportion of protected humans increases, the host-biting rate for the average human decreases. (**B**) The EIR, as a function of intervention coverage, measures the number of infectious bites per person per year. We see the community effects of both ITNs and IRS with DDT, because increasing coverage reduces the EIR for both protected and unprotected humans. At any coverage level, IRS with DDT is not as effective as the use of ITNs, which are not as effective as IRS with bendiocarb, in reducing transmission. We again see that coverage over 80% of IRS with bendiocarb interrupts transmission. This figure appears in color at www.ajtmh.org.

We show the effects of increasing the coverage level of the three interventions, applied singly, on the six field-measurable quantities in Figures 4 and 5. The figures show that IRS with bendiocarb interrupts transmission at coverage levels above 80%. ITNs provide the best personal protection, and IRS with DDT provides good personal protection, whereas IRS-BC provides the best community protection.

We also model increasing coverage of a second intervention in a population with high coverage of a pre-existing vector-control intervention. For each of these simulations, we divide the human population into four groups. Group 1 is humans protected by both interventions. Group 2 is humans protected only by the pre-existing first intervention. Group 3 is humans protected only by the second intervention. Group 4 is unprotected humans.

We then model increasing coverage of the second intervention by moving people from Group 4 to Group 3 and from Group 2 to Group 1. We assume that the second intervention is distributed proportionately so that the probability of a human receiving the intervention is not affected by whether the human is already protected by the pre-existing intervention.

**Figure 6.**
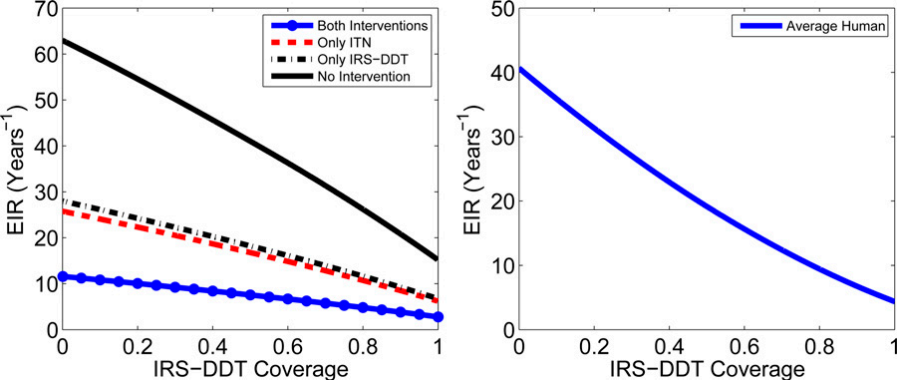
The EIR, measured as infectious bites per person per year, versus coverage of IRS with DDT in a population with a pre-existing ITN coverage level of 60% in an epidemiological setting based on Namawala, Tanzania, with baseline and intervention-modified parameter values described in the Appendix. The figure on the right shows the EIR for an average member of the human population, whereas the figure on the left shows the EIR for each intervention group. When IRS coverage is 0%, 40% of the human population is unprotected, and 60% is protected by ITNs. As the IRS coverage increases, the unprotected humans move to the group that is protected only by IRS, and the ITN users move to the group that is protected by both interventions. Finally, at 100% IRS coverage, 40% of the human population is protected only by IRS with DDT, and 60% is protected by both ITNs and IRS. We see that ITNs provide slightly better personal protection than IRS with DDT, because humans protected by only ITNs have a lower EIR than humans protected by only IRS with DDT. This figure appears in color at www.ajtmh.org.

**Figure 7.**
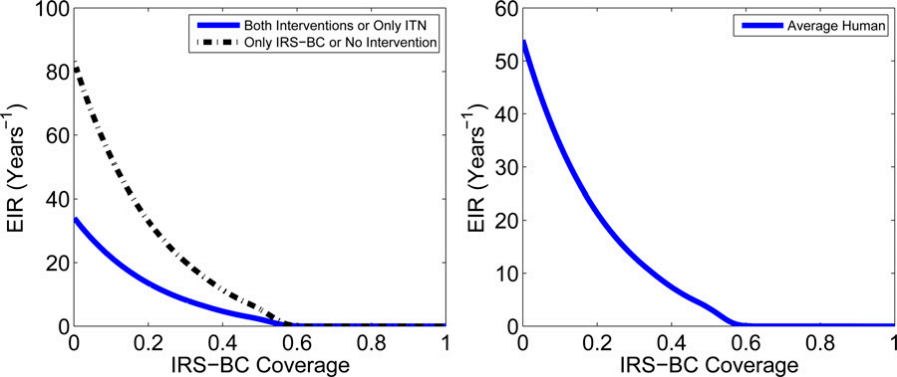
The EIR, measured as infectious bites per person per year, versus coverage of IRS with bendiocarb in a population with a pre-existing ITN coverage level of 60% in an epidemiological setting based on Namawala, Tanzania, with baseline and intervention-modified parameter values described in the Appendix. The figure on the right shows the EIR for an average member of the human population, whereas the figure on the left shows the EIR for each intervention group. Because IRS with bendiocarb does not provide any personal protection (it does not repel or kill mosquitoes before they bite) but only community protection, it does not directly reduce the EIR of a user. Thus, at any coverage level of IRS-BC, humans protected only by IRS-BC have the same EIR as unprotected humans, and humans protected by both IRS-BC and ITNs have the same EIR as humans protected by only ITNs. When IRS coverage is 0%, 40% of the human population is unprotected, and 60% is protected by ITNs. As the IRS coverage increases, the unprotected humans move to the group that is protected only by IRS, and the ITN users move to the group that is protected by both interventions. Finally, at 100% IRS coverage, 40% of the human population is protected only by IRS with bendiocarb, and 60% is protected by both ITNs and IRS. We see strong community effects of IRS-BC with interruption of transmission with coverage above 70%. We note that although the combination of ITNs and IRS with bendiocarb improves control, interruption of transmission occurs at a similar level of IRS-BC coverage as when it is used on its own. This figure appears in color at www.ajtmh.org.

**Figure 8.**
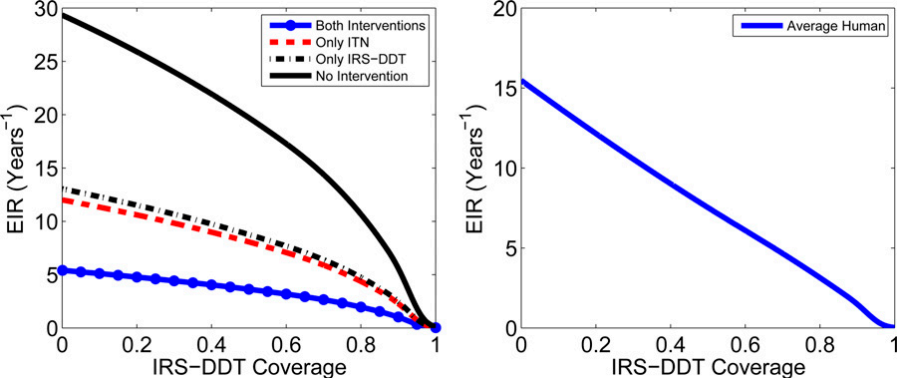
The EIR, measured as infectious bites per person per year, versus coverage of IRS with DDT in a population with a pre-existing ITN coverage level of 80% in an epidemiological setting based on Namawala, Tanzania, with baseline and intervention-modified parameter values described in the Appendix. The figure on the right shows the EIR for an average member of the human population, whereas the figure on the left shows the EIR for each intervention group. When IRS coverage is 0%, 20% of the human population is unprotected, and 80% is protected by ITNs. As the IRS coverage increases, the unprotected humans move to the group that is protected only by IRS, and the ITN users move to the group that is protected by both interventions. Finally, at 100% IRS coverage, 20% of the human population is protected only by IRS with DDT, and 80% is protected by both ITNs and IRS. We see the community effects of IRS, because increasing coverage reduces the EIR for all groups, including the unprotected humans. We also see that ITNs provide slightly better personal protection than IRS with DDT. Because IRS-DDT coverage approaches 100%, EIR approaches 0, and therefore, very high coverage of ITNs and IRS-DDT can substantially reduce or even interrupt transmission. This figure appears in color at www.ajtmh.org.

**Figure 9.**
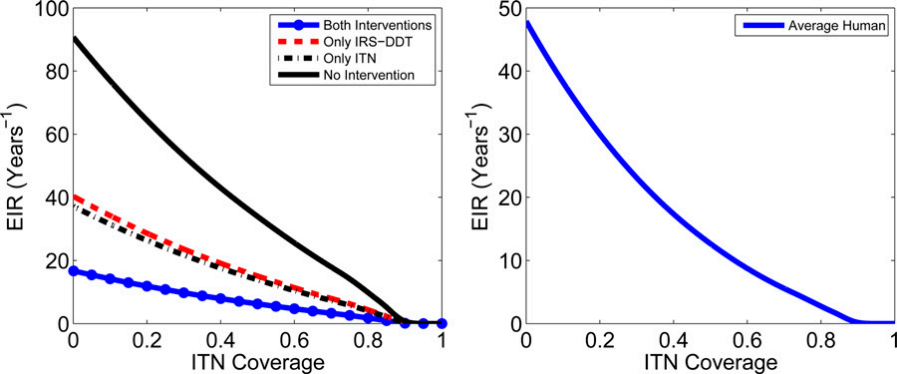
The EIR, measured as infectious bites per person per year, versus coverage of ITNs in a population with a pre-existing coverage level of IRS with DDT of 85% in an epidemiological setting based on Namawala, Tanzania, with baseline and intervention-modified parameter values described in the Appendix. The figure on the right shows the EIR for an average member of the human population, whereas the figure on the left shows the EIR for each intervention group. When ITN coverage is 0%, 15% of the human population is unprotected, and 85% is protected by IRS-DDT. As the ITN coverage increases, the unprotected humans move to the group that is protected only by ITNs, and the humans protected by IRS-DDT move to the group that is protected by both interventions. Finally, at 100% ITN coverage, 15% of the human population is protected only by ITNs, and 85% is protected by both ITNs and IRS. We see the community effects of ITNs, because increasing coverage reduces the EIR for all groups, including the unprotected humans. We also see that ITNs provide slightly better personal protection than IRS with DDT. Similar to Figure 8, we see that very high coverage levels of both ITNs and IRS-DDT can interrupt transmission. This figure appears in color at www.ajtmh.org.

In Figures 6–9, we show and describe the effects of increasing coverage levels of IRS with DDT and with bendiocarb to a population with a pre-existing ITN coverage level of 60%, IRS with DDT to a population with a pre-existing ITN coverage level of 80%, and ITNs to a population with a pre-existing IRS-DDT coverage level of 85%.

We note that the results presented here are from equilibrium analysis, where we assume that enough time has passed to enable the system to reach a stable state that it no longer leaves. We study the properties of the stable fixed point of the system at different coverage levels. It is reasonable to expect, as shown here, the fixed point of the system to depend continuously on the coverage level: increasing the coverage of nets from 20% to 21% or from 70% to 71% would not produce an abrupt change in EIR (or in prevalence or incidence). This is different from time-series data, such as the number of malaria cases in Zanzibar,^14^ where there was a sharp drop after ITN coverage was significantly increased in a short time. In our plots, that would be similar to comparing the EIR at (for example) 20% coverage to the EIR at 70% or 80% coverage, where one would see such an abrupt change. Here, we do not show the transient dynamics of the reduction in transmission over time after the introduction of vector-control interventions.

In other simulations (not shown here), we modeled targeting the population, such as first giving the second intervention to those humans who are already protected by the pre-existing intervention or first giving the second intervention to the unprotected humans. These suggested, as would be expected, that it is more effective to cover the unprotected population first before adding a second intervention to those who are already protected.

## Discussion

We showed interruption of transmission with high coverage of IRS with bendiocarb, agreeing with data from ref. 13 that found no sporozoite-positive mosquitoes in huts sprayed with bendiocarb. However, the part of this data used to parameterize the values for IRS with bendiocarb, as described in the Appendix, is based only on pre-spray and post-spray collections, so it may tend to overestimate the effect of bendiocarb. Furthermore, it is based only on a collection of indoor mosquitoes, thus ignoring the effects of outdoor biting and resting mosquitoes. If there were a subpopulation of completely exophilic mosquitoes, these would continue to transmit malaria despite the spraying of houses. If the same mosquitoes were partially exophilic, IRS with bendiocarb would be less effective in killing them. Although reality would probably be somewhere between these two extremes, it would still be more difficult to achieve interruption of transmission than the model shows with these parameter values. Also, because bendiocarb has a shorter half-life than DDT, it requires more frequent spraying than DDT to retain its effectiveness unless the transmission season is short.

Our simulations also suggest that the effects of IRS with DDT include substantial personal protection and are closer to those of ITNs than to IRS with bendiocarb. Although neither are capable of interrupting transmission on their own in our simulations, our models show that high-coverage levels of both interventions simultaneously can interrupt transmission, even in the high-transmission baseline scenario.

However, when interpreting the results of these simulations, it is important to note the many assumptions that have been made in defining the model and assigning parameter values. We have ignored temporal dynamics and assumed that the daily mosquito emergence rate, *N_v_*_0_, is constant. This ignores seasonal variation, and so, the model mainly applies to settings with perennial, relatively constant transmission. It also means that *N_v_*_0_ is independent of the number of eggs laid. This assumption is valid, given the density-dependence of the development of larval stages, but it breaks down when the adult population is small.^15^

Although we do not explicitly model additional mortality when a mosquito is forced to rest a day before continuing host-seeking, this additional mortality, which would be plausible, is equivalent to increasing the mosquito mortality rate while host-seeking, μ*_vA_*. However, we ignore nonlinear effects such as increased mortality resulting from prolonged lack of blood meals. We also do not model the age structure of mosquitoes, and therefore, older mosquitoes have the same death rate as younger mosquitoes. Whereas the assumption of an exponential death rate means that the number of old mosquitoes is small, it is the older mosquitoes that transmit malaria, and so, we intend to expand the model to include age structure.

We make the simplifying assumption that a mosquito bites only one time in a feeding cycle and that the time, *τ*, needed to complete a feeding cycle after biting does not depend on the type of host. We also ignore nonlinear effects in host-seeking, such as a mosquito being more likely to encounter a particular type of host if it has successfully bitten that type of host before.

We will address many of these assumptions by fully integrating a periodic version of this mosquito transmission model from ref. 1 with the model for malaria in humans described in ref. 2. Allowing the mosquito-emergence rate and the human infectivity to mosquitoes to vary periodically will allow us to capture seasonal variation in mosquito populations and malaria transmission. In addition, we can modulate the daily mosquito-emergence rate as a function of the size of the adult population. Replicating the equations for the mosquito population and transmission dynamics will allow us to model multiple mosquito species, or subgroups, feeding on the same human population.

This full integration will allow us to capture human heterogeneity down to an individual level where a mosquito's probability of surviving and getting infected from a bite on a particular human depends on the characteristics of that human. We can capture transient dynamics such as the decay of nets and insecticide over time. We can also model different delivery strategies for the interventions, such as the timing of IRS campaigns relative to the malaria season and distribution of ITNs through, for example, ante-natal clinics or mass campaigns. This integration will allow us to directly relate the effects of the interventions on morbidity and mortality and to perform cost-effectiveness analysis.

In the simulations described here, we have used the model to determine the effects of the different intervention strategies at different coverage levels. However, we can also use the model from ref. 1 or the fully integrated model to ask the question of what combinations of interventions we would require to achieve certain objectives, including elimination, in different epidemiological settings.

In the analysis of the mathematical model in ref. 1 and in our description here, we have not defined the basic reproductive number, *R*_0_, which is commonly used in mathematical epidemiology to determine a threshold condition for the tendency of a disease to persist in a population. The reason is that a proper definition for *R*_0_ would require a model for the full malaria cycle through humans and mosquitoes, and we have only presented a mathematical model for malaria in mosquitoes. We will address this issue when the two models are fully integrated.

Many of these assumptions and results also need to be corroborated with field data and field studies. A knowledge of exophagy and exophily is important for every location where we would like to apply the model. A comparison of indoor and outdoor biting could be made through indoor and outdoor human-landing catches or newer and more ethical tent traps.^16^ Some degree of exophily after endophagy could be measured through exit window traps. Other assumptions, such as the lack of senescence, can be validated or further explored with insectary experiments by measuring age dependence of mortality of laboratory mosquitoes.

Furthermore, whereas there have been many trials on the effectiveness of ITNs,^17^–^22^ there have been few detailing the effectiveness of IRS^23^ or comparing ITNs with IRS,^24^ and no randomized control trials, so far, have investigated the combinations of the two interventions. Because some countries are beginning to consider this combination^25^ (This refers to Box 2.4 in ref. 25), it is important to investigate when and how this combination would be beneficial with field data.

In malaria-control programs, combinations of IRS and ITNs would probably be of the greatest interest in two perspectives: (1) where the combination can achieve interruption of transmission when either intervention alone cannot, and (2) if the combination is more cost-effective for reduction of the malaria burden than either intervention alone.

Based on these considerations and the results of our model, combination could be tried out in a variety of circumstances. For such research, we would propose the following principles.

• Increasing priority should be given to trials where IRS with an insecticide having high lethality against local vectors is combined with ITNs.
• Prospective studies should be done rigorously with community randomization and a minimum of three arms: IRS, ITNs, and combination. Our results could be applied in sample-size calculation, because the expected differences in EIR from the model could be translated to expected differences in prevalence (although incidence should also be measured).
• It should be attempted to achieve as high coverage rates with both methods as practically possible, to maintain over the long term in a control program.
• In areas with moderate levels of transmission (EIR below 100) where, for example, replastering constrains the effect of IRS, it would be of interest to conduct such trials with close entomological monitoring over all seasons. It might also be considered to examine the results of letting people within a given community choose between IRS and ITNs.
• In areas with intense transmission, the conduct of such a trial would be most rational if it can be expected that both methods can be implemented at high coverage over transmission seasons in the long term.
• It would not be feasible to implement such controlled trials in many operational and epidemiological settings. Therefore, the model results should also be validated against observational studies in areas where IRS and ITNs are combined, for programmatic reasons such as Bioko Island (which recorded substantial decreases in malaria mortality).^26^

Many of the outstanding questions in malaria vector control, such as those listed by Hawley and others,^22^ are difficult or impossible to address with empirical research. Mathematical models provide one approach to some otherwise intractable issues, such as understanding the likely long-term effects of large-scale deployment of interventions that have population-level effects like ITNs and IRS. Large-scale controlled trials of such interventions can never be continued long enough to reach the equilibria that we analyze in this study. Field trials are unlikely to have sufficient power for testing population-level effects of small modifications to established interventions such as changing insecticides. Randomized trials of whether distinct vector-control interventions act synergistically with each other or with other interventions, such as intermittent preventive treatment or prompt and effective treatment, may be prohibitively difficult for both ethical and logistic reasons. However, all these effects can be assessed using mathematical models.

Although we present preliminary results here that are meant as an introduction to the kind of questions that modeling can ask and answer in combining interventions for malaria control and elimination, the models that we propose can also be extended to help define target product profiles for interventions that have not yet reached the field, such as transgenically modified mosquitoes and transmission-blocking vaccines, or to assess the potential of older methods, such as source reduction, that may be difficult to implement on a large scale. We plan to soon present a fully integrated model for malaria in humans and mosquitoes that will include realistic health-systems settings to examine combinations of vector-control interventions with other malaria-control interventions and provide further outputs, such as effectiveness in reducing morbidity and mortality and cost-effectiveness analysis.

## Supplementary Material

Appendix Mathematical Description of Model

## Figures and Tables

**Table 1:** Description of the parameters of the mosquito malaria transmission model (modified from table 1 in ref. 1)

Parameter	Description
*T*	The length of each time step. For this model, we fix *T* = 1 day (dimension: time).
*n*	Number of different types of hosts.
*m*	Number of different types of hosts that are susceptible to malaria (*m* ≤ *n*).
*N_v0_*	The emergence rate (per day) of new mosquitoes (dimension: animals × time^−1^; N_v0_ > 0).
*N_i_*	Total number of hosts of type i (dimension: animals; N_i_ > 0).
α_i_	Availability rate of each host of type i to mosquitoes. This rate includes the reduction in availability of a host because of diversion (dimension: animals^−1^ × time^−1^; α_i_ > 0).
μ_vA_	Per capita mosquito death rate while searching for a blood meal (dimension: time^−1^; μ_vA_ > 0).
θ_d_	Maximum length of time that a mosquito searches for a host in 1 day if it is unsuccessful (dimension: time; 0 < θ_d_ < *T*).
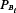	Probability that a mosquito bites a host of type i after encountering a host of type i (0 < 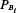 < 1).
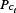	Probability that a mosquito finds a resting place after biting a host of type i (0 < 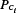 < 1).
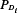	Probability that a mosquito survives the resting phase after biting a host of type i (0 < 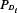 < 1).
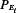	Probability that a mosquito lays eggs and returns to host-seeking after biting a host of type i (0 < 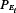 < 1).
τ	Time required for a mosquito that has encountered a host to return to host-seeking (provided that the mosquito survives to search again). It is the partial duration of the feeding cycle, excluding the time that it takes the mosquito to find a host. We assume that τ is equal to the resting period of the mosquito (i.e., the time needed to digest blood and produce eggs; dimension: time).
θ_s_	Duration of the extrinsic incubation period. This is the time from ingestion of gametocytes until sporozoites are present in the salivary glands (dimension: time; θ_s_ ≥ τ).
K_vi_	Probability of parasite transmission from a host of type i to render a susceptible mosquito infective, per bite, provided that the mosquito survives long enough. (This term includes the probability that the parasite then survives in the mosquito to produce sporozoites; 0 ≤ K_vi_ < 1; K_vi_ = 0 for i representing non-humans).

**Table 2:** Description of selected derived parameters of the mosquito malaria transmission model (selected and modified from table 2 in ref. 1)

Parameter	Description
*P_A_*	Probability that a mosquito does not find a host and does not die in 1 night of searching.
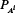	Probability that a mosquito finds a host of type i on a given night.
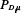	Probability that a mosquito dies while resting after biting a host of type i, where 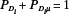 .
*P_df_*	Probability that a mosquito finds a host on a given night and then successfully completes the feeding cycle.
*P_dif_ :*	Probability that a mosquito finds a host on a given night, gets infected, and then successfully completes the feeding cycle.

**Table 3:** Baseline parameter values for the simulations

Parameter	Dimension	Baseline value
*N_v_*_0_	Animals × Days^−1^	25,000
α*_i_*	Animals^−1^ × Days^−1^	0.0072
μ*_vA_*	Days^−1^	1.6
θ*_d_*	Days	0.33
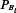	Dimensionless	0.95
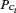	Dimensionless	0.95
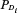	Dimensionless	0.99
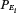	Dimensionless	0.88
τ	Days	3
θ*_s_*	Days	11

The details of the parameter descriptions are in Table 1. As described in ref. 1, we set the time step to one day (*T* = 1). The number of different types of hosts and hosts susceptible to malaria is *n* = *m* = 2 for simulations where there is only intervention and *n* = *m* = 4 for simulations where we combine two interventions. Although the population size of each type of human, *N_i_*, varies depending on the coverage level, we keep the total population size fixed at 1,000 (i.e., ). The human infectivity to mosquitoes, *K_vi_*, is determined as described in the Appendix.

**Table 4:** Effects of interventions on baseline parameters

Parameter	ITNs	IRS-DDT	IRS-BC
α*_i_*	0.56	0.44	1
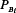	0.73	1	1
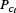	0.73	1	1
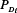	1	0.76	0.19

We define the intervention effect such that the intervention-modified parameter is the product of the intervention effect and the baseline parameter value. For example, an intervention effect value of 1 implies no effect on the parameter and 0 implies full effect.

## References

[1] Chitnis N Smith T Steketee R 2008 A mathematical model for the dynamics of malaria in mosquitoes feeding on a heterogeneous host population J Biol Dyn 2 259 285 10.1080/17513750701769857 22876869

[2] Smith T Maire N Ross A Penny M Chitnis N Schapira A Studer A Genton B Lengeler C Tediosi F de Savigny D Tanner M 2008 Towards a comprehensive simulation model of malaria epidemiology and control Parasitology 135 1507 1516 18694530 10.1017/S0031182008000371

[3] Saul AJ Graves PM Kay BH 1990 A cyclical feeding model for pathogen transmission and its application to determine vector capacity from vector infection rates J Appl Ecol 27 123 133

[4] Saul A 2003 Zooprophylaxis or zoopotentiation: the outcome of introducing animals on vector transmission is highly dependent on the mosquito mortality while searching Malar J 32 10.1186/1475-2875-2-32 PMC222927 14565850

[5] Le Menach A Takala S McKenzie FE Perisse A Harris A Flahault A Smith DL 2007 An elaborated feeding cycle model for reductions in vectorial capacity of night-biting mosquitoes by insecticide-treated nets Malar J 6 10.1186/1475-2875-6-10 PMC1794417 17254339

[6] Killeen GF Smith TA 2007 Exploring the contributions of bed nets, cattle, insecticides and excitorepellency in malaria control: a deterministic model of mosquito host-seeking behaviour and mortality Trans R Soc Trop Med Hyg 101 867 880 17631372 10.1016/j.trstmh.2007.04.022 PMC1949412

[7] Killeen GF Ross A Smith T 2006 Infectiousness of malaria-endemic human populations to vectors Am J Trop Med Hyg 75 (Suppl 2) 38 45 16931814 10.4269/ajtmh.2006.75.2_suppl.0750038

[8] van den Berg H Takken W 2009 Evaluation of integrated vector management Trends Parasitol 25 71 76 19110470 10.1016/j.pt.2008.11.005

[9] Service MW 1993 Mosquito Ecology. Field Sampling Methods2nd ed London, United Kingdom Elsevier

[10] Garrett-Jones C Grab B 1964 The assessment of insecticidal impact on the malaria mosquito's vectorial capacity, from data on the population of parous females Bull World Health Organ 31 71 86 14230896 PMC2555159

[11] Smith T Killeen GF Maire N Ross A Molineaux L Tediosi F Hutton G Utzinger J Dietz K Tanner M 2006 Mathematical modeling of the impact of malaria vaccines on the clinical epidemiology and natural history of *Plasmodium falciparum* malaria: overview Am J Trop Med Hyg 75 (Suppl 2) 1 10 16931810 10.4269/ajtmh.2006.75.2_suppl.0750001

[12] Smith A Webley DJ 1968 A verandah-trap hut for studying the house-frequenting habits of mosquitoes and for assessing insecticides. III. The effect of DDT on behaviour and mortality Bull Entomol Res 59 33 46 4390166 10.1017/s000748530000300x

[13] Sharp BL Ridl FC Govender D Kuklinski J Kleinschmidt I 2007 Malaria vector control by indoor residual insecticide spraying on the tropical island of Bioko, Equatorial Guinea Malar J 6 10.1186/1475-2875-6-52 PMC1868751 17474975

[14] Bhattarai A Ali AS Kachur SP Martensson A Abbas AK Khatib R Al-Mafazy AW Ramsan M Rotllant G Gerstenmaier JF Fabrizio M Abdulla S Montgomery SM Kaneko A Björkman A 2007 Impact of artemisinin-based combination therapy and insecticide-treated nets on malaria burden in Zanzibar PLoS Med 4 e309 17988171 10.1371/journal.pmed.0040309 PMC2062481

[15] Gimnig JE Ombok M Otieno S Kaufman MG Vulule JM Walker ED 2002 Density-dependent development of *Anopheles gambiae* (Diptera: Culicidae) larvae in artificial habitats J Med Entomol 39 162 172 11931252 10.1603/0022-2585-39.1.162

[16] Govella NJ Chaki PP Geissbuhler Y Kannady K Okumu F Charlwood JD Anderson RA Killeen GF 2009 A new tent trap for sampling exophagic and endophagic members of the *Anopheles gambiae* complex Malar J 8 10.1186/1475-2875-8-157 PMC2720981 19602253

[17] D'Alessandro U Olaleye BO McGuire W Langerock P Bennett S Aikins MK Thomson MC Cham MK Cham BA Greenwood BM 1995 Mortality and morbidity from malaria in Gambian children after introduction of an impregnated bednet programme Lancet 345 479 483 7861874 10.1016/s0140-6736(95)90582-0

[18] Nevill CG Some ES Mung'Ala VO Mutemi W New L Marsh K Lengeler C Snow RW 1996 Insecticide-treated bednets reduce mortality and severe morbidity from malaria among children on the Kenyan coast Trop Med Int Health 1 139 146 8665377 10.1111/j.1365-3156.1996.tb00019.x

[19] Binka FN Kubaje A Adjuik M Williams LA Lengeler C Maude GH Armah GE Kajihara B Adiamah JH Smith PG 1996 Impact of permethrin impregnated bednets on child mortality in Kassena-Nankana district, Ghana: a randomized controlled trial Trop Med Int Health 1 147 154 8665378 10.1111/j.1365-3156.1996.tb00020.x

[20] Habluetzel A Diallo DA Esposito F Lamizana L Pagnoni F Lengeler C Traore C Cousens SN 1997 Do insecticide-treated curtains reduce all-cause child mortality in Burkina Faso? Trop Med Int Health 2 855 862 9315044 10.1046/j.1365-3156.1997.d01-413.x

[21] Takken W 2002 Do insecticide-treated bednets have an effect on malaria vectors? Trop Med Int Health 7 1022 1030 12460393 10.1046/j.1365-3156.2002.00983.x

[22] Hawley WA ter Kuike FO Steketee RS Nahlen BL Terlouw DJ Gimnig JE Shi YP Vulule JM Alaii JA High-tower AW Kolczak MS Kariuki SK Phillips-Howard PA 2003 Implications of the western Kenya permethrin-treated bed net study for policy, program implementation, and future research Am J Trop Med Hyg 68 (Suppl 4) 168 173 12749501

[23] Pluess B Tanser FC Lengeler C Sharp BL 2010 Indoor residual spraying for preventing malaria Cochrane Database of Systematic Reviews 4 CD006657 doi:10.1002/14651858.CD006657.pub2 10.1002/14651858.CD006657.pub2 PMC6532743 20393950

[24] Guyatt HL Corlett SK Robinson TP Ochola SA Snow RW 2002 Malaria prevention in highland Kenya: indoor residual house-spraying vs. insecticide-treated bednets Trop Med Int Health 7 298 303 11952944 10.1046/j.1365-3156.2002.00874.x

[25] World Health Organization 2008 World Malaria Report 2008 Available at http://malaria.who.int/wmr2008/malaria2008.pdf Accessed May 2010

[26] Kleinschmidt I Schwabe C Benavente L Torrez M Ridl FC Segura JL Ehmer P Nchama GN 2009 Marked increase in child survival after four years of intensive malaria control Am J Trop Med Hyg 80 882 888 19478243 PMC3748782

[27] Killeen GF Kihonda J Lyimo E Oketch FR Kotas ME Mathenge E Schellenberg JA Lengeler C Smith TA Drakeley CJ 2006 Quantifying behavioural interactions between humans and mosquitoes: evaluating the protective efficacy of insecticidal nets against malaria transmission in rural Tanzania BMC Infect Dis 6 10.1186/1471-2334-6-161 PMC1657018 17096840

[28] Evans RG 1993 Laboratory evaluation of the irritancy of bendiocarb, lambda-cyhalothrin and DDT to *Anopheles gambiae* J Am Mosq Control Assoc 9 285 293 8245937

