# Supplementary material for: Comparing the Effectiveness of Malaria Vector-Control Interventions Through a Mathematical Model
Source: Am J Trop Med Hyg. 2010 Aug 5;83(2):230–40. doi: 10.4269/ajtmh.2010.09-0179 (PMC2911164; doi:10.4269/ajtmh.2010.09-0179)
Supplement: Appendix Mathematical Description of Model [file SD1.pdf]

APPENDIX

MATHEMATICAL DESCRIPTION OF MODEL

Here, we summarize the main equations from ref. 1. The system of equations for the mosquito malaria transmission model, with parameters and derived parameters described in Tables 1 and 2, respectively, is [1, (3)],

$$N_v(t) = N_{v0}T + P_A N_v(t-1) + P_{df} N_v(t-\tau), \tag{1a}$$

$$O_v(t) = P_{dif}(N_v(t-\tau) - O_v(t-\tau)) + P_A O_v(t-1) + P_{df} O_v(t-\tau), \tag{1b}$$

$$\begin{aligned} S_v(t) = {} & P_{dif}\left(\sum_{j=0}^{k_+}\binom{\theta_s-(j+1)\tau+j}{j} P_A^{\theta_s-(j+1)\tau} P_{df}^j\right) \\ & \times (N_v(t-\theta_s) - O_v(t-\theta_s)) \\ & + \left[\sum_{l=1}^{\tau-1} P_{dif}\left(\sum_{j=0}^{k_{l+}}\binom{(\theta_s+l)-(j+2)\tau+j}{j}\right.\right. \\ & \left. \times P_A^{(\theta_s+l)-(j+2)\tau} P_{df}^j\right) P_{df} \\ & \left. \times \left(N_v\left(t-(\theta_s+l)\right) - O_v\left(t-(\theta_s+l)\right)\right)\right] \\ & + P_A S_v(t-1) + P_{df} S_v(t-\tau), \end{aligned} \tag{1c}$$

with

$$k_+ = \left\lfloor \frac{\theta_s}{\tau} \right\rfloor - 1 \text{ and } k_{l+} = \left\lfloor \frac{\theta_s + l}{\tau} \right\rfloor - 2, \tag{2}$$

where $\lfloor x \rfloor$ is the floor function, that is, the greatest integer less than x; and the binomial coefficient is

$$\binom{a}{b} = \frac{a!}{b!(a-b)!}.$$

The derived parameters are,[1]

$$P_A = e^{-\left(\sum_{i=1}^{n}\alpha_i N_i + \mu_{vA}\right)\theta_d},$$

$$P_{A^i} = \left(1 - e^{-\left(\sum_{k=1}^{n}\alpha_k N_k + \mu_{vA}\right)\theta_d}\right) \times \frac{\alpha_i N_i}{\sum_{k=1}^{n}\alpha_k N_k + \mu_{vA}}, \tag{3}$$

$$P_{df} = \sum_{i=1}^{n} P_{A^i} P_{B_i} P_{C_i} P_{D_i} P_{E_i},$$

$$P_{dif} = \sum_{i=1}^{n} P_{A^i} P_{B_i} P_{C_i} P_{D_i} P_{E_i} K_{vi}.$$

The system (1) has a unique fixed point [1, (6)],

$$N_v^* = \frac{N_{v0}T}{1 - P_A - P_{df}}, \tag{4a}$$

$$O_v^* = \frac{N_v^* P_{dif}}{1 - P_A - (P_{df} - P_{dif})}, \tag{4b}$$

$$\begin{aligned} S_v^* = {} & \frac{P_{dif}(N_v^* - O_v^*)}{1 - P_A - P_{df}} \times \left[\left(\sum_{j=0}^{k_+}\binom{\theta_s-(j+1)\tau+j}{j} P_A^{\theta_s-(j+1)\tau} P_{df}^j\right)\right. \\ & \left. + \sum_{l=1}^{\tau-1}\left(\sum_{j=0}^{k_{l+}}\binom{(\theta_s+l)-(j+2)\tau+j}{j} P_A^{(\theta_s+l)-(j+2)\tau} P_{df}^{j+1}\right)\right], \end{aligned} \tag{4c}$$

with k_+ and k_{l+} defined in (2). This fixed point is globally asymptotically stable if all the eigenvalues of the evolution matrix (Υ in ref. 1) of the system of equations (1) are inside the unit circle. While we could not show this analytically for all parameter values,[1] we numerically verified that the magnitudes of all eigenvalues of Υ for each of our simulations here were less than 1.

For this globally asymptotically stable fixed point, we calculate the field-measurable quantities,[1]

Parous rate:

$$M = \frac{P_{df}}{1 - P_A}$$

Delayed oocyst rate:

$$o_v = \frac{P_{dif}}{1 - P_A - (P_{df} - P_{dif})}$$

Sporozoite rate:

$$\begin{aligned} s_v = {} & \frac{P_{dif}}{1 - P_A - (P_{df} - P_{dif})} \times \left[\left(\sum_{j=0}^{k_+}\binom{\theta_s-(j+1)\tau+j}{j} P_A^{\theta_s-(j+1)\tau} P_{df}^j\right)\right. \\ & \left. + \sum_{l=1}^{\tau-1}\left(\sum_{j=0}^{k_{l+}}\binom{(\theta_s+l)-(j+2)\tau+j}{j} P_A^{(\theta_s+l)-(j+2)\tau} P_{df}^{j+1}\right)\right] \end{aligned}$$

Host-biting rate:

$$\sigma_i = \frac{N_{v0}}{N_i} \frac{P_{A^i} P_{B_i}}{1 - P_A - P_{df}}$$

Entomological Inoculation Rate (EIR):

$$\Xi_i = \frac{N_{v0}}{N_i} \times \frac{P_{A^i} P_{B_i}}{1 - P_A - P_{df}} \frac{P_{dif}}{1 - P_A - (P_{df} - P_{dif})}$$
$$\times \left[\left(\sum_{j=0}^{k_+} \binom{\theta_s - (j+1)\tau + j}{j} P_A^{\theta_s - (j+1)\tau} P_{df}^j \right) + \sum_{l=1}^{\tau-1} \left(\sum_{j=0}^{k_{l+}} \binom{(\theta_s + l) - (j+2)\tau + j}{j} P_A^{(\theta_s + l) - (j+2)\tau} P_{df}^{j+1} \right) \right]$$

Vectorial capacity:

$$\Gamma = \frac{N_{v0}}{\sum_{k=1}^{n} N_k} \times \frac{\left(\sum_{i=1}^{n} P_{A^i} P_{B_i} \right) P_{df}}{(1 - P_A - P_{df})^2}$$
$$\times \left[\left(\sum_{h=0}^{k_+} \binom{\theta_s - (h+1)\tau + h}{h} P_A^{\theta_s - (h+1)\tau} P_{df}^h \right) + \sum_{l=1}^{\tau-1} \left(\sum_{h=0}^{k_{l+}} \binom{(\theta_s + l) - (h+2)\tau + h}{h} P_A^{(\theta_s + l) - (h+2)\tau} P_{df}^{h+1} \right) \right].$$

DETAILS OF THE HUMAN INFECTIVITY TO MOSQUITOES AS A FUNCTION OF EIR

We let x represent the EIR (measured per person per year) and $f(x)$ represent the human infectivity to mosquitoes as a function of EIR. We require a realistic $f(x)$ that captures the biology of malaria transmission through humans and the simulation results in Figure 3 to have the following properties:

1. The domain is $[0, \infty]$.
2. The range is $[0, f^*]$ for some $f^* < 1$.
3. $f(x)$ is smooth (continuous and differentiable) over the domain, that is, $f(x) \in C^1([0, \infty])$.
4. $f(0) = 0$.
5. There exists an x^* with $f(x^*) = f^*$, such that,

$$f'(x) > 0 \text{ for } x < x^*,$$
$$f'(x) = 0 \text{ for } x = x^*,$$
$$f'(x) < 0 \text{ for } x > x^*.$$

6. $f''(x) < 0$ for $0 \le x \le x^*$.
7. There exists an $\bar{f} < f^*$ such that

$$\lim_{x \to \infty} f(x) = \bar{f}.$$

We define $f(x)$ using piecewise rational functions as,

$$f(x) = \begin{cases} f_1(x) & 0 \le x \le x^* \\ f_2(x) & x > x^* \end{cases}, \qquad (5)$$

where,

$$f_1(x) = \frac{mx(x-a)}{x-b},$$

$$f_2(x) = \frac{hx^2(x-g)(x-j)}{(x-l)^4}.$$

Ensuring that $f_1(x^*) = f_2(x^*)$ and $f_1'(x^*) = f_2'(x^*) = 0$ provides,

$$x^* = \frac{(3(g+j)l - 2gj) + \sqrt{(3(g+j)l - 2gj)^2 - 8gjl(4l - g - j)}}{2(4l - g - j)},$$
$$a = \frac{b^2 - (b - x^*)^2}{b},$$
$$m = \frac{f_2(x^*)(x^* - b)}{x^*(x^* - a)}.$$

We fit the rest of the parameters in (5) to the model simulations in Figure 3 using least squares to provide,

$$b = 240.44,$$
$$g = 2.3565,$$
$$h = 0.046406,$$
$$j = 2.5935,$$
$$l = 1.4970,$$

so that $f(x)$ (5) satisfies all seven properties.

PARAMETER VALUES AND THE EFFECTS OF INTERVENTIONS

We use baseline parameter values in the absence of interventions (except for P_{D_i} and P_{E_i} which we discuss in the section on IRS with DDT) from ref. 1, which are based largely on data from Killeen and Smith[27] for *Anopheles gambiae* in Namawala, Tanzania. We assume that each intervention affects a certain number of mosquito survival and infection parameters (that we describe below) while other parameters remain unchanged by the intervention. Thus some parameter values change depending on whether a type of host has or does not have an intervention. We note again that N_i is the number of hosts of type i and varies depending on the coverage level of the intervention, and not on the biological properties of the intervention itself.

Except for parameters that are defined as natural numbers, we use two significant figures for their values. The parameter values used for each individual intervention are given in Table 4. Parameter values for simulations of combined interventions are calculated as described below.

Insecticide-treated nets. The effects of ITNs were modeled in ref. 1 and we use the same parameter values here. The availability rate of ITN users is reduced by 44% and the survival probability of a mosquito biting a human is reduced by 46%, divided equally between before and after biting. Using the notation, i = 1 representing ITN users and i = 2 unprotected humans, we model the effects of ITNs as,

$$\alpha_1 = 0.56\alpha_2,$$
$$P_{B_1} = 0.73P_{B_2},$$
$$P_{C_1} = 0.73P_{C_2},$$

with,

$$P_{D_1} = P_{D_2},$$
$$P_{E_1} = P_{E_2}.$$

Indoor residual spraying with DDT. We assume that IRS with DDT deters mosquitoes from entering houses, thus reducing the human availability rate, α_i, and kills resting mosquitoes after they rest on walls, thus reducing the mosquito's probability of surviving the resting phase, P_{D_i}, but does not affect any of the other parameters in the model. We use data from Smith and Webley[12] to determine the numerical values for these reductions. In this subsection, we use the notation of $i = 1$ to denote humans in houses sprayed with DDT and $i = 2$ to denote unprotected humans.

Table IX[12] shows the deterrent effect of DDT on *Anopheles gambiae* in the Magugu area of Tanzania over each month since treatment of the hut. Since the model contains only constant parameters, we average the deterrent effect over all months to give a value of 56%. We assume this corresponds to a 56% decrease in the probability that a mosquito encounters a human protected by IRS-DDT, P_{A^i},

$$P_{A^1} = 0.44 P_{A^2}.$$

Assuming $N = 2$ and $N_1 = N_2$ from[12] (the number of inhabitants in the treated and untreated experimental huts were the same) in (3), some algebraic manipulations show that,

$$\alpha_1 = \alpha_2 P_{A^1} / P_{A^2},$$

so,

$$\alpha_1 = 0.44\alpha_2.$$

Table VI[12] shows the number of *An. gambiae* counted and collected in treated and untreated verandah-trap huts in Magugu for every month since treatment. The table divides the mosquitoes by feeding status and whether they were dead or alive. We assume that the proportion of dead fed mosquitoes out of all fed mosquitoes is the probability of a mosquito dying while resting. Averaged over all months, we find that for huts treated with IRS, $P_{D_{1\mu}} = 0.25$ and for untreated huts, $P_{D_{1\mu}} = 0.0064$. Thus,

$$P_{D_1} = 0.75,$$
$$P_{D_2} = 0.99,$$
$$P_{D_1} = 0.76 P_{D_2},$$

that is, IRS with DDT decreases the mosquito's probability of surviving the resting phase by 24%.

As ref. 1 primarily investigated the effectiveness of ITNs, the exact values of P_{D_i} and P_{E_i} did not matter as long as the product matched the probability of surviving a feeding cycle in the absence of intervention, P_f. We now use this data from Smith and Webley[12] to set P_{D_i} in the absence of IRS and choose a corresponding value of P_{E_i} that matches the original P_f,

$$P_{E_i} = 0.88.$$

We thus use $P_{D_i} = 0.99$ and $P_{E_i} = 0.88$ for the baseline values in the absence of an intervention.

Indoor residual spraying with bendiocarb. Laboratory studies by Evans (1993)[28] on *Anopheles gambiae* showed that the irritancy effect of bendiocarb was statistically similar to that of distilled water. We assume in our simulations that IRS with bendiocarb does not repel mosquitoes and thus does not reduce the availability rate of humans in houses sprayed with bendiocarb. Using the notation in this subsection of $i = 1$ for humans in houses sprayed with bendiocarb and $i = 2$ for unprotected humans, we thus have,

$$\alpha_1 = \alpha_2.$$

We use data from Sharp et al. (2007)[13] to determine the effects of bendiocarb (*Ficam*™) on mosquito mortality. They collected data on *An. gambiae* s.s. on Bioko island on the number of mosquitoes and their sporozoite rates in sentinel huts, before and after treatment. They list the number of mosquitoes collected per trap per 100 nights in Table 1 as 23.9 before treatment and 1.9 after treatment. In the absence of more detailed information on the mosquitoes, we assume that all mosquitoes caught have survived the resting phase.

Since we are considering two separate time points, we assume there is only one type of host, and label the time before spraying as $(j) = 2$ and the time after spraying as $(j) = 1$. The number of mosquitoes caught per night[1] is,

$$x^{(j)} = \frac{N_{\upsilon 0}}{1 - P_A - P_{A^1} P_{B_1} P_{C_1} P_{D_1}^{(j)} P_{E_1}} P_{A^1} P_{B_1} P_{C_1} P_{D_1}^{(j)}. \quad (6)$$

We substitute (6) into the ratio of $x^{(1)}$ to $x^{(2)}$ to solve for $P_{D_1}^{(1)}$,

$$P_{D_1}^{(1)} = \frac{(1 - P_A) x^{(1)} P_{D_1}^{(2)}}{\left(1 - P_A - P_{A^1} P_{B_1} P_{C_1} P_{D_1}^{(2)} P_{E_1}\right) x^{(2)} + \left(P_{A^1} P_{B_1} P_{C_1} P_{D_1}^{(2)}\right) x^{(1)}}.$$

Substituting $x^{(1)} = 1.9$, $x^{(2)} = 23.9$, $P_{D_1}^{(2)} = 0.99$, and the baseline values for other parameters,

$$P_{D_1}^{(1)} = 0.19.$$

Thus to model the effects of IRS with bendiocarb on mosquito mortality, we use,

$$P_{D_1} = 0.19 P_{D_2}.$$

Parameter values for two concurrent interventions. When modeling humans with multiple interventions, we assume that the effects of the interventions are independent and cumulative. For example, humans that are protected by both ITNs and IRS with DDT, the availability rate of the protected human will be 0.56×0.44 times the availability rate of the unprotected humans. The mosquito survival probabilities, P_{B_i} and P_{C_i} will decrease by a factor of 0.73 as defined for ITNs, and P_{D_i} will decrease by a factor of 0.76 as defined for IRS-DDT.

Box 1

Glossary of Modeling Terms

System: A set or arrangement of things so related or connected as to form an integrated whole. Example: The malaria infection and illness "system" where the malaria parasite, mosquito vector, human host and their determinants and interactions establish the pattern of malaria infection and illness in the population.
Deterministic System: A system in which no randomness is present and any given starting state at a given point in time always leads to the same consecutive states.
Dynamical System: A deterministic system that is governed by a fixed rule which determines the evolution of the state over time: any given state always leads to a unique consecutive state.
Stochastic System: A stochastic system is one that includes randomness or noise.
Differential Equations: A system of differential equations is a system based on continuous values of the independent variable, usually time (for example, time, t, is a real number greater than zero). The equations define the rate at which the variables of the system change at a given time, t, as functions of the variables at time, t.
Difference Equations: A system of difference equations is a system based on discrete values of the independent variable, usually time (for example, time, t, is an integer greater than or equal to zero: $t = 0, 1, 2, 3, \ldots$). The equations define the variables of the system at a given time, t, as functions of the variables at the previous time step, $t - 1$.
Agent-Based/Individual-Based Model: A simulation or computational model composed of a number of agents or individuals that interact (usually stochastically) within a network. A significant advantage of agent-based models as opposed to traditional differential equation or difference equation models is that they allow the incorporation of substantially more detail and complexity. However, this means that the models are computationally intensive. Furthermore, they lack the mathematical theory and analytical techniques developed over the last century for traditional dynamical systems models.
